# Factors Associated with the Decline of Kidney Function Differ among eGFR Strata in Subjects with Type 2 Diabetes Mellitus

**DOI:** 10.1155/2012/687867

**Published:** 2012-12-18

**Authors:** Shu Meguro, Masuomi Tomita, Yusuke Kabeya, Takeshi Katsuki, Yoichi Oikawa, Akira Shimada, Toshihide Kawai, Hiroshi Itoh, Yoshihito Atsumi

**Affiliations:** ^1^Nephrology, Endocrinology and Metabolism Division, Department of Internal Medicine, School of Medicine, Keio University, Tokyo 160-8582, Japan; ^2^Department of Internal Medicine, Saiseikai Central Hospital, Tokyo 108-0073, Japan

## Abstract

*Introduction*. There is no report about risk factors for renal deterioration according to the clinical stage, divided by the estimated glomerular filtration rate (eGFR) in type 2 diabetes. *Materials and Methods*. We evaluated the factors correlated with the annual eGFR decline in 1303 subjects with type 2 diabetes whose eGFR was ≥30 mL/min/1.73 m^2^. eGFR strata were defined by baseline eGFR value as follows: stratum 1: ≥90, stratum 2: ≥60, <90, and stratum 3: ≥30, <60. *Results*. The annual eGFR decline was 2.3 ± 5.4 mL/min/1.73 m^2^ in overall subjects. Multiple linear regression analysis demonstrated that age, male sex, systolic blood pressure, logarithmically transformed albumin excretion rate (AER), eGFR strata, and hemoglobin concentration were significantly correlated with the annual eGFR decline. When stratified by eGFR, the factors that showed a significant correlation were different among eGFR strata. AER was significantly correlated with annual eGFR decline in all eGFR strata. Hemoglobin concentration showed a significant correlation only in the advanced eGFR stratum. *Conclusion*. The factors correlated with the annual eGFR decline were different among eGFR strata in type 2 diabetes mellitus, and hemoglobin concentration and AER were important factors for renal deterioration, especially in the advanced eGFR stratum.

## 1. Introduction

Diabetic kidney disease is the leading cause of end-stage renal disease (ESRD), and the number of individuals requiring chronic dialysis or renal transplantation is still increasing in most countries [1]. Both urinary albumin excretion and estimated glomerular filtration rate (eGFR) are independently associated with the worsening of kidney function [2, 3]. We previously reported that a very high proportion of Japanese subjects with type 2 diabetes mellitus with stage 3 chronic kidney disease (CKD) progressed to stage 4 CKD within 3 years, and that the existence of albuminuria further accelerated the clinical course [3]. Although the clinical course of the deterioration of kidney function might differ among study populations, another study from the United Kingdom reconfirmed a similar relation between albuminuria and eGFR [4]. We also found that hemoglobin concentration was a significant predictor of deterioration to CKD stage 4 [3].

Anemia is a common complication of chronic kidney disease (CKD). In diabetes mellitus, anemia develops earlier and is more severe than in patients with renal impairment with other causes [5, 6]. Anemia in diabetes mellitus is known to be a risk factor for the development and progression of micro- and macrovascular complications of diabetes mellitus as well as increased mortality independent of the presence or severity of diabetic nephropathy [7]. A reduced hemoglobin concentration, even within the normal range, is associated with an increased risk of ESRD and death in subjects with chronic renal failure [8]. It was also reported that lower hemoglobin level was related to subsequent decline of kidney function in subjects with persistent macroalbuminuria [9] and also in those without albuminuria [10]. However, there is no report on the risk factors for decline of kidney function according to clinical stage, divided by eGFR. Especially, data on the GFR decline in subjects with type 2 diabetes mellitus whose GFR was less than 60 mL/min/1.73 m^2^ are scarce despite the clinical importance of this stage in relation to further deterioration of kidney function.

We prospectively evaluated the significance of hemoglobin concentration and other clinical factors in the three-year decline of kidney function in a hospital-based study cohort.

## 2. Materials and Methods

### 2.1. Subjects

The details of the study participants were described previously [3]. All of the subjects admitted to Saiseikai Central Hospital for a 2-week diabetic education program from January 1999 to December 2004 were study candidates. The study was conducted in accordance with the Declaration of Helsinki. Those subjects whose eGFR was less than 30 mL/min/1.73 m^2^ and those with a strong suspicion of renal disease with causes other than diabetic nephropathy (e.g., glomerulonephritis) were excluded. Of the 1901 subjects, 7 died (5 due to cancer, 1 sudden death, and 1 acute myocardial infarction), 38 moved to other medical facilities for medical or personal reasons, and 553 withdrew for unknown reasons. As a result, we analyzed 1303 Japanese subjects with type 2 diabetes mellitus whose eGFR was ≥30 mL/min/1.73 m^2^. The subjects were instructed on a standard life-style modification according to the recommendations of the Japan Diabetes Society (JDS).

### 2.2. Clinical Data Collection

Blood collection after an overnight fast was performed in the early morning of Day 2 of hospitalization. Serum fasting glucose, total cholesterol, HDL cholesterol, triglycerides, and serum creatinine were determined by enzymatic methods, and several other biochemical assays were performed with autoanalyzers. Serum creatinine concentration three years after admission was evaluated in the outpatient clinic. Data obtained soon after the end of the three-year period were adopted. Almost all subjects were evaluated within four months after the end of the three-year period. HbA1c level on admission was determined by high-performance liquid chromatography (HPLC: Arkray Inc., Kyoto, Japan) according to the recommended method by (JDS) at that time and converted to the National Glycohemoglobin Standardization Program (NGSP) value [11].

Twenty-four-hour urine collection was performed from Day 2 to Day 3 of hospitalization. Microalbuminuria was defined as albumin excretion rate (AER) in 24-hour urine collection of 20 to 200 *μ*g/min and macroalbuminuria as more than 200 *μ*g/min. eGFR (mL/min/1.73 m^2^) was calculated as 194 × Cr^−1.094^ × Age^−0.287^ (with further multiplication by 0.739 for female subjects) using the equation provided by the Japanese Society of Nephrology [12]. The study subjects were subdivided by eGFR level, as follows: stratum 1: eGFR_baseline_≧90 mL/min/1.73 m^2^, stratum 2 : 60 ≦eGFR_baseline_ < 90 mL/min/1.73 m^2^, and stratum 3: 30 ≦eGFR_baseline_ < 60 mL/min/1.73 m^2^. Annual GFR decline was calculated by the formula: (eGFR_baseline_ − eGFR_after  3  years_)/3. Annual percent reduction of eGFR from the baseline value was calculated by the formula: annual GFR decline/eGFR_baseline_  × 100. All subjects underwent funduscopic examination by trained ophthalmologists during or just before admission.

### 2.3. Statistical Analysis

Data are expressed as mean ± standard deviation (SD) or number (%) except for triglyceride and AER. Data of triglyceride and AER are expressed as median and interquartile range. Differences in continuous variables among the three groups were tested by ad hoc Fisher's least significant difference (LSD) multiple comparison method after the analysis of variance (ANOVA). Differences in frequencies of each group were investigated by chi-squared test. Multiple linear regression analysis with forced entry method was performed to detect independent significant predictors of decline of kidney function. Some variables were excluded from this analysis because of collinearity among variables. As AER showed a skewed distribution, we used the common logarithm of values. Stratified analysis of risk factors for GFR reduction by eGFR strata was needed, as the annual GFR reductions were different among eGFR strata. Two-tailed *P* < 0.05 was considered statistically significant. All analyses were performed using IBM SPSS software ver. 18.0 (SPSS Inc., an IBM company, Japan).

## 3. Results

### 3.1. Baseline Characteristics of Subjects

Baseline characteristics of the subjects are shown in Table 1. The mean age of this study cohort was around 60 years, two-thirds were male, and mean duration of diabetes was about ten years. Mean systolic blood pressure was 132 mmHg and mean diastolic blood pressure 75 mmHg. Mean HbA1c was 9.1%. About 30% of study participants had diabetic retinopathy. Over 70% of hypertensive subjects and around 90% of hypertensive subjects with macroalbuminuria were being treated with renin-angiotensin system (RAS) blockers.

Several baseline characteristics were significantly different among eGFR strata. Briefly, subjects with advanced eGFR stratum were older, predominantly male, and had longer disease duration, higher systolic blood pressure, better glycemic control, higher AER, and lower hemoglobin level compared to those with less advanced eGFR strata.

### 3.2. Factors That Affect Annual eGFR Decline

The annual GFR decline was 2.3 ± 5.4 mL/min/1.73 m^2^ in overall subjects. That in male subjects was 2.5 ± 5.4 and that in female subjects was 2.0 ± 5.4 mL/min/1.73 m^2^, with no significant sex difference. Annual GFR decline was 1.5 ± 4.9, 2.9 ± 5.0, and 7.1 ± 7.5 mL/min/1.73 m^2^ in subjects with normoalbuminuria, microalbuminuria, and macroalbuminuria, respectively. Annual percent reduction of eGFR was 1.4 ± 5.5, 3.2 ± 5.7, and 9.8 ± 8.8% in subjects with normoalbuminuria, microalbuminuria, and macroalbuminuria, respectively. Annual GFR decline was significantly greater in stratum 1 compared to the other strata, and when we analyzed the annual percent reductions of eGFR, the differences between stratum 1 and the other strata and also between stratum 2 and stratum 3 were significant (Table 1).

The results of multiple linear regression analysis of annual eGFR decline are shown in Table 2. Age, male sex, systolic blood pressure, logarithmically transformed AER, eGFR strata, and hemoglobin concentration were significantly correlated with annual eGFR decline. The existence of diabetic retinopathy showed a borderline association with the annual GFR reduction. eGFR strata showed the highest standard regression coefficient value.

### 3.3. Multiple Linear Regression Analysis Stratified by eGFR

The results of multiple linear regression analysis of the annual eGFR decline stratified by eGFR strata are shown in Table 3. It revealed that factors that were significantly correlated with the annual eGFR decline were different among the eGFR strata. AER at baseline was significantly correlated with the annual eGFR decline in all eGFR strata. Systolic blood pressure showed a significant correlation with the annual eGFR decline only in stratum 2. The existence of diabetic retinopathy was not correlated with the annual eGFR decline in all eGFR strata. HbA1c at baseline was significantly associated with the annual eGFR decline in stratum 1 and 2 but not in stratum 3. On the other hand, the hemoglobin concentration showed a significant correlation with the annual eGFR decline only in stratum 3, but this correlation was relatively strong (partial regression coefficient: −0.249, *P* < 0.01). Male sex was also correlated with the annual GFR decline in stratum 3.

## 4. Discussion

It has been confirmed that both the amount of urinary albumin and eGFR value are useful to predict the outcome of kidney function in subjects with type 2 diabetes [2–4]. Also, as the decline of GFR is finally the most important clinical marker for the development of ESRD, several risk factors for the decline of GFR have been advocated. Albuminuria/proteinuria, glycemic control, blood pressure control, existence of diabetic retinopathy, and the existence of anemia are proposed representative risk factors [2–4, 9, 10]. We demonstrated that annual eGFR decline rate and the factors correlated with the eGFR reduction varied according to the baseline eGFR status in this study.

Annual percent reduction of eGFR from the baseline value showed a U shape pattern, being higher in stratum 1 and stratum 3 than in stratum 2. The steeper decline of eGFR in stratum 1 might reflect the hyperfiltration state in type 2 diabetes [13, 14].

The degree of albuminuria was correlated with the annual eGFR decline in all eGFR strata in our study. On the other hand, systolic blood pressure was correlated with the annual eGFR decline only in stratum 2. The fact that over 70% of hypertensive patients were treated with antihypertensive agents that were predominantly RAS blockers in our study might have influenced the study results.

HbA1c at baseline was significantly associated with the annual eGFR decline in the less advanced eGFR strata but not in stratum 3. As multiple linear regression analysis of stratum 3 subjects revealed that HbA1c was not correlated with the annual eGFR decline, the effect of reduced hemoglobin concentration on HbA1c value in accordance with the deterioration of kidney function was negligible. We consider that these results suggest the importance of earlier blood glucose control in preventing diabetic nephropathy, although this observational study cannot provide a definite conclusion about therapy.

The existence of diabetic retinopathy did not relate to the annual eGFR decline. As logarithmically transformed AER was significantly higher in subjects with retinopathy compared to those without, the relation of retinopathy to worsening of kidney function might be confounded by albuminuria. On the other hand, although there is a common mechanism of diabetic microvascular disease in retinopathy and nephropathy, diabetic kidney disease in type 2 diabetes mellitus might be influenced by various factors other than hyperglycemia compared to retinopathy. At least, from the fact that over half of subjects with stratum 3 in this study did not have any sign of diabetic retinopathy, the absence of diabetic retinopathy did not imply a benign renal outcome in subjects with type 2 diabetes with an advanced eGFR stratum.

On the contrary, hemoglobin concentration did not show an association with the annual eGFR decline in less advanced eGFR strata. However, the correlation between hemoglobin concentration and the annual eGFR decline in stratum 3 has a strong clinical significance, because eGFR decline has a greater clinical impact in patients with a more advanced eGFR stratum. It has been reported that anemia develops earlier in diabetes [5, 6], and anemia in diabetes mellitus is related to a worse outcome of vascular complications as well as increased mortality [7–10]. A previous study showed that hemoglobin concentration was correlated with the worsening of kidney function with or without albuminuria [9, 10]. However, in our study, a correlation of hemoglobin concentration with eGFR decline was observed only in the reduced eGFR group, independent of albuminuria. This may imply that the etiology of the hemoglobin reduction is linked to the pathophysiology of the eGFR reduction, rather than that of urinary albumin excretion. The main cause of anemia in diabetes is suspected to be reduced erythropoietin production relative to the degree of anemia, and some researchers observed the microscopic injury of the renal tubulointerstitium, where erythropoietin is produced, in diabetic subjects [15, 16]. It has been revealed that the decline of kidney function in CKD was more strongly correlated with tubular and interstitial changes than with glomerular changes [15], and our results corresponded well with this finding if the reduced hemoglobin concentration in the advanced eGFR stratum reflected renal tubulointerstitial damage. Although it was reported that pathologic damage of the tubulointerstitium was found relatively early in the clinical course, hemoglobin concentration was not correlated with the eGFR decline in the less advanced stratum in our study, but was significantly correlated only in stratum 3. However, we consider it reasonable that advanced tubulointerstitial damage, which gives rise to organ dysfunction such as reduced hemoglobin concentration, corresponds to advanced eGFR stratum to some extent.

Yokoyama et al. recently reported the risk factors for eGFR decline in association with the progression of albuminuria in Japanese type 2 diabetes [14]. Their study was a clinic-based cohort study with an excellent design. The significance of albuminuria in eGFR decline and that of HbA1c in earlier stages corresponded to our results, but the effects of the existence of retinopathy and anemia on eGFR decline were different. We do not know how the differences arose, but the fact that our study population included subjects of a more advanced stage compared to their study might have influenced the results. In addition, the limitations of each study might have affected their results.

A limitation of our study is that it was an observational study performed at a single hospital in Japan. The drop-out rate was considerably high. As we demonstrated in our prior publication [3], the dropout subjects were dominantly male, younger with a shorter duration of diabetes mellitus, with a higher ratio of smokers, lower serum creatinine level, and less frequent history of CVD. So the higher dropout rate could have made the disease progression rate in this study higher than the real incidence. Annual eGFR decline was computed only from two points. Baseline data obtained just after admission might reflect poor glycemic control and high blood pressure prior to admission, which might have affected the GFR and AER levels in our study. AER determined in this study was a single measurement, although it is usually recommended to evaluate it on three different days. Moreover, as is customary with observational studies, we could not distinguish an observed association as a cause or effect.

The heterogeneity of type 2 diabetic mellitus is another possible limitation. Similar previous reports in terms of the progression of kidney dysfunction have been performed in various situations. One study was performed in Caucasian subjects with macroalbuminuria, in which subjects with retinopathy comprised 70% of the total [9]. A Japanese study excluded subjects with macroalbuminuria and did not mention retinopathy [10]. The degree and prevalence of possible risk factors as well as other diabetic complications in the study populations might influence the study results, so we should be cautious in comparing and interpreting the results of different study populations.

## 5. Conclusion

We reported that the factors associated with the annual eGFR decline might be different among eGFR strata in subjects with type 2 diabetes mellitus, and that hemoglobin concentration as well as AER was a very important clinical factor to predict the worsening of kidney function, especially in stratum 3. Further understanding of how each clinical marker such as albuminuria, eGFR decline, and the reduction of hemoglobin concentration reflects the renal pathology is important.

## Figures and Tables

**Table 1:** Baseline characteristics of subjects data are mean  SD. AER and TG were expressed as median and interquartile range and were transformed to the common logarithm for calculations. *P < 0.05, **P < 0.01, and ***P < 0.001 versus stratum 1, ^#^ *P* < 0.05, ^##^ *P* < 0.01, and ^###^ *P* < 0.001 versus stratum 2 as determined by multiple comparison method (Fisher's LSD) after ANOVA. BMI: body mass index, SBP: systolic blood pressure, DBP: diastolic blood pressure, FPG: fasting plasma glucose, TC: total cholesterol, HDL-C: HDL cholesterol, TG: triglyceride, UA: uric acid, Cr: creatinine, and AER: albumin excretion rate.

Parameter	Overall (*n* = 1303)	Stratum 1 (*n* = 432)	Stratum 2 (*n* = 740)	Stratum 3 (*n* = 131)	*P* value
Age (years)	60.0 ± 8.9	56.0 ± 8.3	61.3 ± 8.6***	65.2 ± 7.6^∗∗∗, ###^	<0.001
BMI (kg/m^2^)	23.6 ± 3.8	23.7 ± 4.1	23.5 ± 3.6	24.5 ± 3.5*	<0.05
Male (%)	68.6	60.0	72.5	75.6	<0.001
Duration of diabetes (y)	10.4 ± 8.3	8.9 ± 7.4	10.7 ± 8.3**	12.9 ± 9.7^∗∗∗, ##^	<0.001
Diabetic retinopathy (%)	28.9	26.4	28.3	42.0	<0.01
SBP (mmHg)	132.0 ± 17.6	131.5 ± 18.9	131.5 ± 16.4	137.3 ± 18.9^∗∗, ###^	<0.01
DBP (mmHg)	74.5 ± 10.8	74.6 ± 11.6	74.5 ± 10.4	74.2 ± 10.1	n.s.
FPG (mmol/L)	9.7 ± 2.9	10.4 ± 3.0	9.4 ± 2.7***	8.8 ± 2.8^∗∗∗, #^	<0.001
HbA1c (%)	9.1 ± 1.8	9.4 ± 1.9	8.9 ± 1.7***	8.7 ± 1.7***	<0.001
TC (mmol/L)	5.3 ± 1.0	5.4 ± 1.0	5.2 ± 0.9*	5.4 ± 0.9	<0.05
HDL-C (mmol/L)	1.4 ± 0.4	1.4 ± 0.4	1.3 ± 0.4	1.4 ± 0.4	n.s.
TG (mmol/L)	111 (78–163)	109 (79–171)	110 (76–159)*	122 (90–179)^#^	<0.05
UA (mg/dL)	5.2 ± 1.4	4.7 ± 1.3	5.3 ± 1.3***	6.3 ± 1.7^∗∗∗, ###^	<0.001
Cr (mg/dL)	0.7 ± 0.5	0.6 ± 0.8	0.8 ± 0.2***	1.1 ± 0.2^∗∗∗, ###^	<0.001
eGFR (mL/min/1.73 m^2^)	82.7 ± 19.7	106.5 ± 13.3	78.0 ± 9.6***	53.0 ± 12.2^∗∗∗, ###^	<0.001
eGFR after 3 years	75.7 ± 20.5	92.5 ± 19.0	73.7 ± 14.6***	47.8 ± 15.4^∗∗∗, ###^	<0.001
Annual GFR decline (mL/min/1.73 m^2^)	2.3 ± 5.4	4.6 ± 6.5	1.4 ± 4.4***	1.7 ± 5.8***	<0.001
Annual GFR decline (% reduction from baseline)	2.5 ± 6.29	4.2 ± 6.0	1.7 ± 5.6^∗∗∗, #^	2.9 ± 8.9^∗, #^	<0.001
AER (*μ*g/min)	12.5 (5.8–41.3)	11.5 (6.0–29.9)	11.4 (5.6–35.5)	35.4^∗∗∗, ###^ (10.3–232.5)	<0.001
Hemoglobin (g/L)	143 ± 13	143 ± 14	144 ± 13	137 ± 16^∗∗∗, ###^	<0.001

**Table 2:** Multiple linear regression analysis of annual eGFR decline in all subjects. Multiple linear regression analysis of annual GFR reduction was performed. AER was transformed to the common logarithm for calculations. SBP: systolic blood pressure, AER: albumin excretion rate. Adjusted *R* ^2^ = 0.213, *P* < 0.001.

Parameter	Partial regression coefficient (*β*)	Standard error (S.E.)	*P* value
Age (years)	0.096	0.017	<0.01
Male: 1, female: 0	0.100	0.343	<0.01
SBP (mmHg)	0.073	0.008	<0.01
HbA1c (%)	0.240	0.079	<0.001
Log_10_ AER (*μ*g/min)	0.228	0.212	<0.001
eGFR strata (1–3)	−0.288	−0.244	<0.001
Retinopathy: 1, none: 0	0.052	0.323	0.051
Hemoglobin (g/L)	−0.102	−0.115	<0.01

**Table 3:** Multiple linear regression analysis of annual eGFR decline stratified by eGFR strata. Multiple linear regression analysis of annual eGFR reduction was performed. Adjusted *R* ^2^ and *P* values of multiple regression model of stratum 1–3 were *R* ^2^ = 0.130 and *P* < 0.001, *R* ^2^ = 0.154 and *P* < 0.001, and *R* ^2^ = 0.469 and *P* < 0.001, respectively. AER was transformed to the common logarithm for calculations. SBP: systolic blood pressure, AER: albumin excretion rate.

Parameter		Stratum 1				Stratum 2				Stratum 3	
	*β*	SE	*P* value		*β*	SE	*P* value		*β*	SE	*P* value
Age (years)	0.069	0.039	n.s.		0.153	0.153	<0.001		−0.071	0.040	n.s.
Male: 1, female: 0	0.083	0.754	n.s.		0.119	0.381	<0.01		0.181	0.726	<0.05
SBP (mmHg)	0.045	0.017	n.s.		0.100	0.010	<0.01		0.128	0.017	n.s.
HbA1c (%)	0.272	0.165	<0.001		0.236	0.091	<0.001		0.128	0.170	n.s.
Log_10_ AER (*μ*g/min)	0.185	0.485	<0.001		0.195	0.246	<0.001		0.516	0.388	<0.001
Retinopathy: 1, None: 0	0.085	0.730	n.s.		0.054	0.357	n.s.		−0.075	0.647	n.s.
Hemoglobin (g/L)	−0.086	0.269	n.s.		−0.071	0.132	n.s.		−0.249	0.201	<0.01
